# Cryoablation inhibition of distant untreated tumors (abscopal effect) is immune mediated

**DOI:** 10.18632/oncotarget.24105

**Published:** 2018-01-18

**Authors:** Xueling Yang, Yongfei Guo, Zhi Guo, Tongguo Si, Wenge Xing, Wenwen Yu, Yan Wang

**Affiliations:** ^1^ Department of Interventional Therapy, Tianjin Medical University Cancer Institute and Hospital, Tianjin 300060, China; ^2^ National Clinical Research Center of Cancer, Tianjin 300060, China; ^3^ Tianjin’s Clinical Research Center for Cancer, Tianjin 300060, China; ^4^ Key Laboratory of Cancer Prevention and Therapy, Tianjin 300060, China; ^5^ Department of Interventional Radiology, Tianjin Hospital of ITCWM Nankai Hospital, Tianjin 100020, China; ^6^ Department of Biotherapy, Tianjin Medical University Cancer Institute and Hospital, Tianjin 300060, China

**Keywords:** cryoablation, abscopal effect, immune response

## Abstract

Cryoablation is moderately effective against prostate cancer. Of note, the off-target or enlarged therapeutic effect after cryoablation is reported in routine clinical practice. To uncover it, we constructed a bilateral inguinal transplantation model of prostate cancer. All the mice were randomly subdivided into three groups: Group A (Control group), Group B (Surgery group), and Group C (Cryoablation group). All the procedures in three groups were conducted only for tumors in the target region (right groin). The tumors in untargeted region (left groin) received no treatment. We measured the growth of untargeted tumors and lung metastasis rate, and then explored the changes in a series of immune cells and danger signals. First, our results revealed the protective effect of cryoablation treatment against the abscopal tumor. The possible mechanism was mediated by an increase in the number of CD4^+^ T cells, CD8^+^ T cells, ratio T helper 1 (Th1)/Th2, the killing activity of cytotoxic T lymphocytes and NK cells. Hsp70 may be involved in the modulation of the immune response. The combination of weakened Ki67 activity and activated immune response delayed spectator tumor growth, decreased the pulmonary metastasis rate, and prolonged animal survival, with an inducible abscopal effect.

## INTRODUCTION

Cryoablation is used in a variety of medical treatments with hollow needles (cryoprobes) through which cooled, thermally conductive fluids are circulated. Ablation occurs in tissue that has been frozen by at least three mechanisms: (1) formation of ice crystals within cells thereby disrupting membranes, and interrupting cellular metabolism among other processes; (2) coagulation of blood and interrupting blood flow to the tissue leading to ischemia and cell death; (3) and induction of apoptosis. Cryoablation has been widely used against solid tumors in the lungs, liver, breast, kidneys, and prostate [1–5].

Evidence indicates that cryoablation is moderately effective, especially against early-stage prostate cancer [6, 7]. Moreover, a rare clinical response is noticed in routine clinical practice, which is an off-target or enlarged therapeutic effect after cryoablation. Similar phenomena are also well-recognized during other medical treatments, such as radiotherapy and gene therapy. Two terms the “bystander effect” and “abscopal effect” emerged in this regard. In November 1992, Hatsumi Nagasawa and John B. Little [8] first reported this radiobiological phenomenon (bystander effect), in which unirradiated cells exhibit irradiation effects as a result of signals received from nearby irradiated cells. In suicide gene therapy, the “bystander effect” is the ability of the transfected cells to transfer death signals to neighboring tumor cells [9]. The term “abscopal effect” was coined by Mole in 1953[10]. Unlike the “bystander effect,” “abscopal effect” is a phenomenon where the response to radiation is seen in an organ/site distant from the irradiated organ/area, that is, the responding cells are not juxtaposed with the irradiated cells. T cells and dendritic cells have been implicated in the mechanism of this effect.

**Figure 1:**
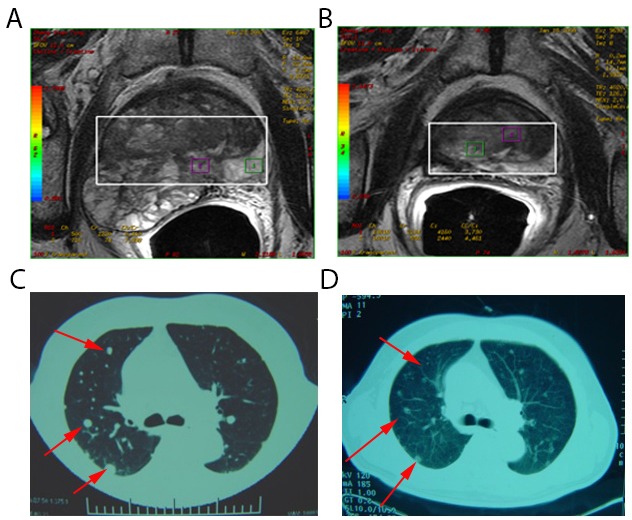
Spontaneous regression of lung metastases after cryoablation for metastatic prostate cancer. **(A)** Pelvic magnetic resonance imaging of a 78-year-old man with metastatic prostate carcinoma (Stage T3bN0M1c). This lesion was treated with cryoablation. **(B)** Pelvic magnetic resonance imaging 1 month post-treatment appeared inactive. **(C)** Computed tomography scans of pulmonary metastasis of the patient before treatment revealed multiple metastases in lung tissues (red arrow). **(D)** Computed tomography scans of pulmonary metastasis of the patient 1 month post-treatment indicated regression of lesions in lung tissues (red arrow).

Here, the abscopal effect was also detected after cryoablation of metastatic cancer where localized treatment causes shrinking of lung metastases outside the scope of the localized treatment (Figure 1). Although this phenomenon is extremely rare, its effect on cancer can be stunning, leading to the disappearance of malignant tumors throughout the entire body. Such success has been described for a variety of cancers, including parietal pleura adenocarcinoma [11], musculoskeletal tumors [12], and papillary fibroelastoma [13]. Nonetheless, scientists are not certain how the abscopal effect works to eliminate cancer in patients. Clinical studies on breast cancer suggest that the effect may depend upon activation of the immune system [14]. In a case study from the Memorial Sloan-Kettering Cancer Center on metastatic melanoma [15], these dynamic changes in tumor-directed antibody levels and immune cell populations were seen during the abscopal effect.

In previous clinical work, we found that there are some patients in whom pulmonary metastases regressed spontaneously after cryoablation for prostate cancer. Subsequent studies have revealed that a cryoimmunological response might participate in this process [16–18]. We speculated that *in vivo* cryoablation of a tumor, alone, can induce an immunostimulatory systemic antitumor response [19], having an effect of an antitumor vaccine. Nevertheless, cryoablation alone may produce an insufficient immune response, depending on various factors. Hence, we speculated that cryoablation may broadly stimulate the immune system to fight abscopal cancer. As a cellular marker for proliferation, its expression is strongly associated with tumor cell proliferation and growth, and is widely used in routine pathological investigation as a proliferation marker [20]. In order to observe the growth of untargeted tumor, we choose to evaluate Ki67 index for proliferative activity except diameter measure. Besides of that, various constitutive or inducible danger signals released by injured cells are known to play a determinant role in alarming the immune system against self-damage [21–23], such as high mobility box 1 protein (HMGB1), heat-shock protein 70 (Hsp70). Our precious animal experience [24] also revealed an rising HMGB1 level after cryoablation for prostate cancer, which indicated that danger signals may play a vital role in the cryo- immunological response.

To validate the abscopal effect of cryoablation and to explore and illustrate the possible mechanism of this phenomenon, we designed this study to investigate the effects of inflammatory and immune mechanisms.

## RESULTS

### Abscopal effects in untreated tumors

Compared to that from Group A (Control group) and Group B (Surgery group), the diameter of tumor in the left region (untargeted region) in group C (Cryoablation group) decreased significantly (*P* < 0.05, *P* < 0.05, respectively) on day 7 and day 14. There was no statistically significant difference (*P* > 0.05) on day 7 between Group B (Surgery group) and Group A (Control group), but slight shrinking in group B (Surgery group) (*P* < 0.05) was seen on day 14 (Table 1).

**Table 1:** The diameter of tumors in the untargeted region in different groups (cm)

	**Group A (Control group)**	**Group B (Surgery group)**	**Group C (Cryoablation group)**
Before treatment	0.5±0.08	0.5±0.08	0.5±0.05
Day 7 after treatment	1.3±0.12^1^	1.4±0.13^1^	0.8±0.15^ab1^
Day 14 after treatment	2.3±0.22^12^	2.0±0.13^a12^	1.2±0.15^ab12^

Note:^a^compared with that of Group A, *P <* 0.05; ^b^compared with that of Group B, *P <* 0.05; ^1^compared with that before treatment, *P <* 0.05; ^2^compared with that on day 7 after treatment, *P <* 0.05.

### Overall survival (OS)

**Figure 2:**
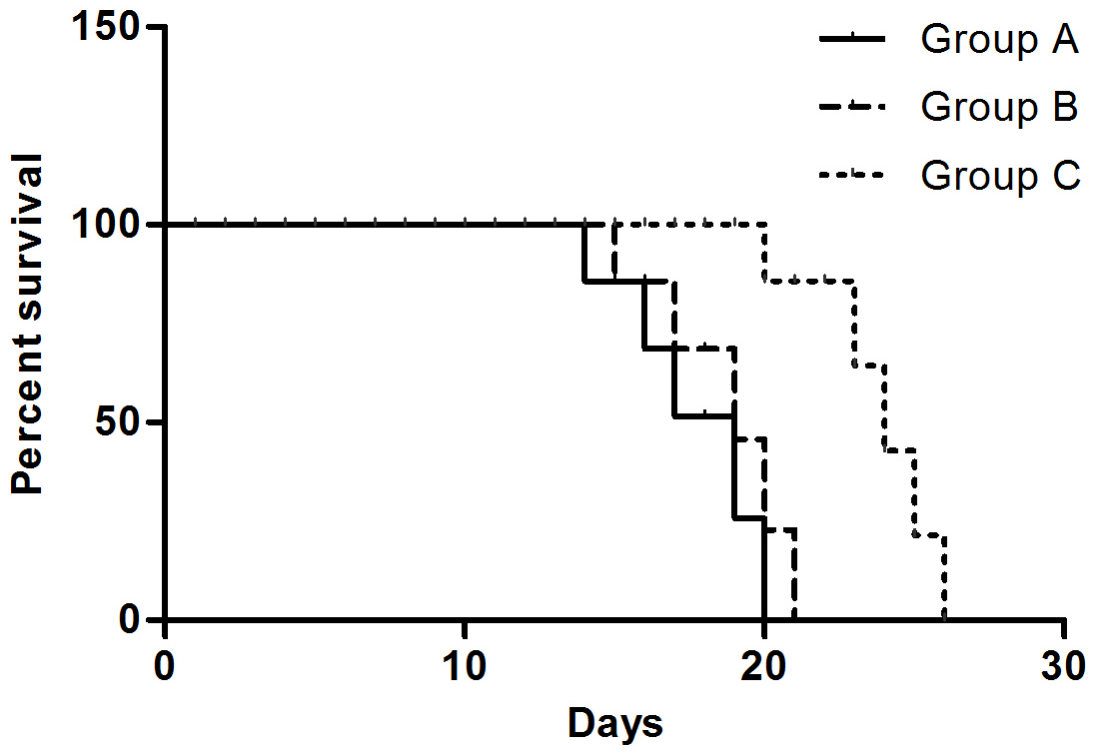
OS of mice in three groups. The median OS of mice in Group A, Group B, and Group C was 19, 19, and 24 days, respectively.

The median OS in three groups was 19, 19, and 24 days respectively (Figure 2). Compared to Group A (Control group) and Group B (Surgery group), the median OS of Group C (Cryoablation group) was significantly prolonged (*P* < 0.001). There was no statistically significant difference (*P* > 0.05) between Groups A and B.

### Lung metastasis positivity rate

**Figure 3:**
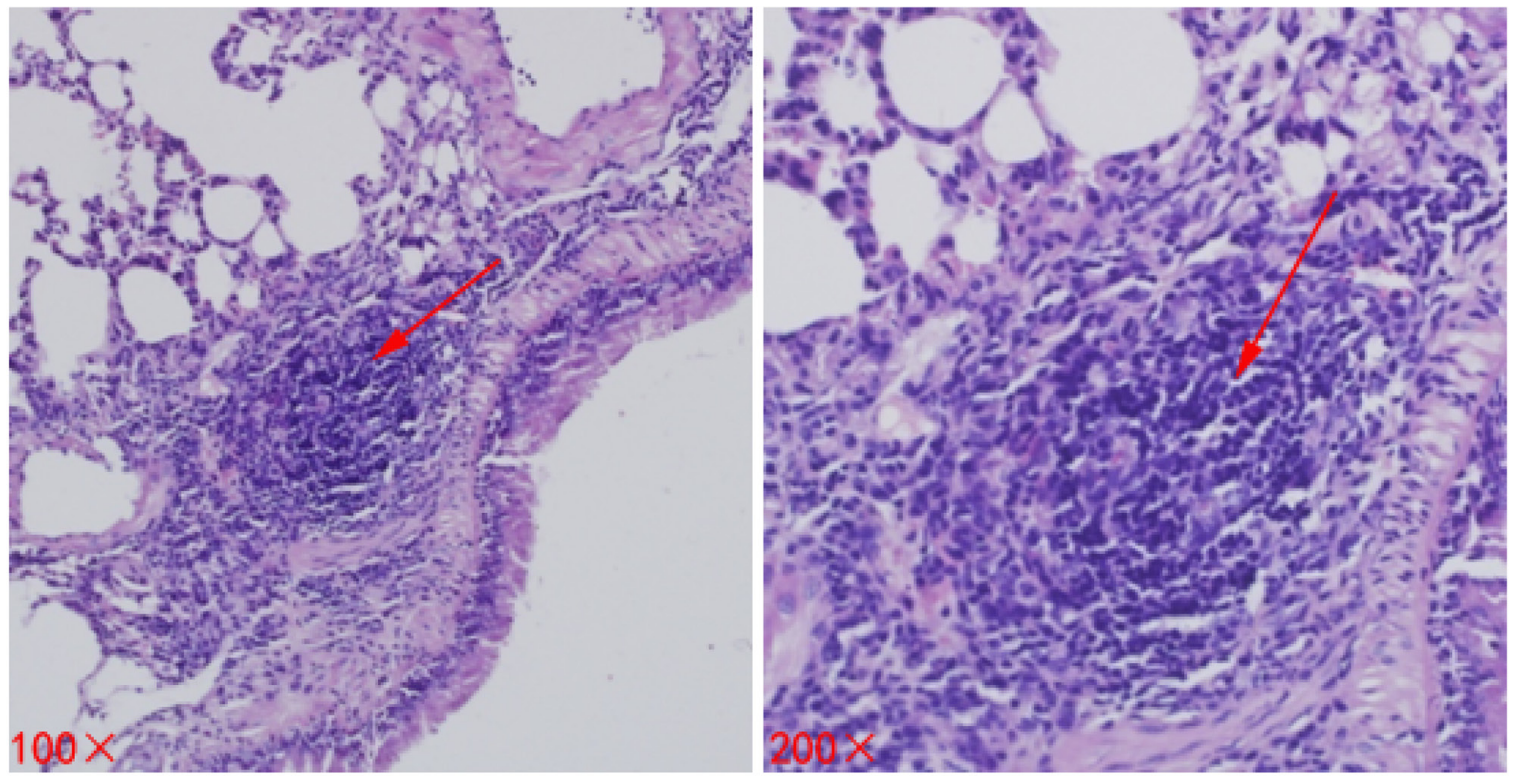
Metastases (lesions) in lung tissues of mice. The lesion is indicated by a red arrow.

As time goes on, the lung metastasis positivity rate showed an increasing trend in each group, there was no significant difference on day 7 (*P* = 0.562). Nonetheless, compared to Group A (Control group) and Group B (Surgery group), lung metastasis positivity rate in the Group C (Cryoablation group) decreased significantly (*P* < 0.05, *P* < 0.05, respectively) on day 14 (Figure 3, Table 2).

**Table 2:** Lung metastasis in mice in different groups (%)

	**Group A (Control group)**	**Group B (Surgery group)**	**Group C (Cryoablation group)**
Before treatment	0% (0/10)	0% (0/10)	0% (0/10)
Day 7 after treatment	20% (2/10)	20% (2/10)	0% (0/10)
Day 14 after treatment	100% (10/10)	80% (8/10)	20% (2/10)

### CD4^+^ T cell, CD8^+^ T cell, and natural killer (NK) cell proportions in the spleen

**Figure 4:**
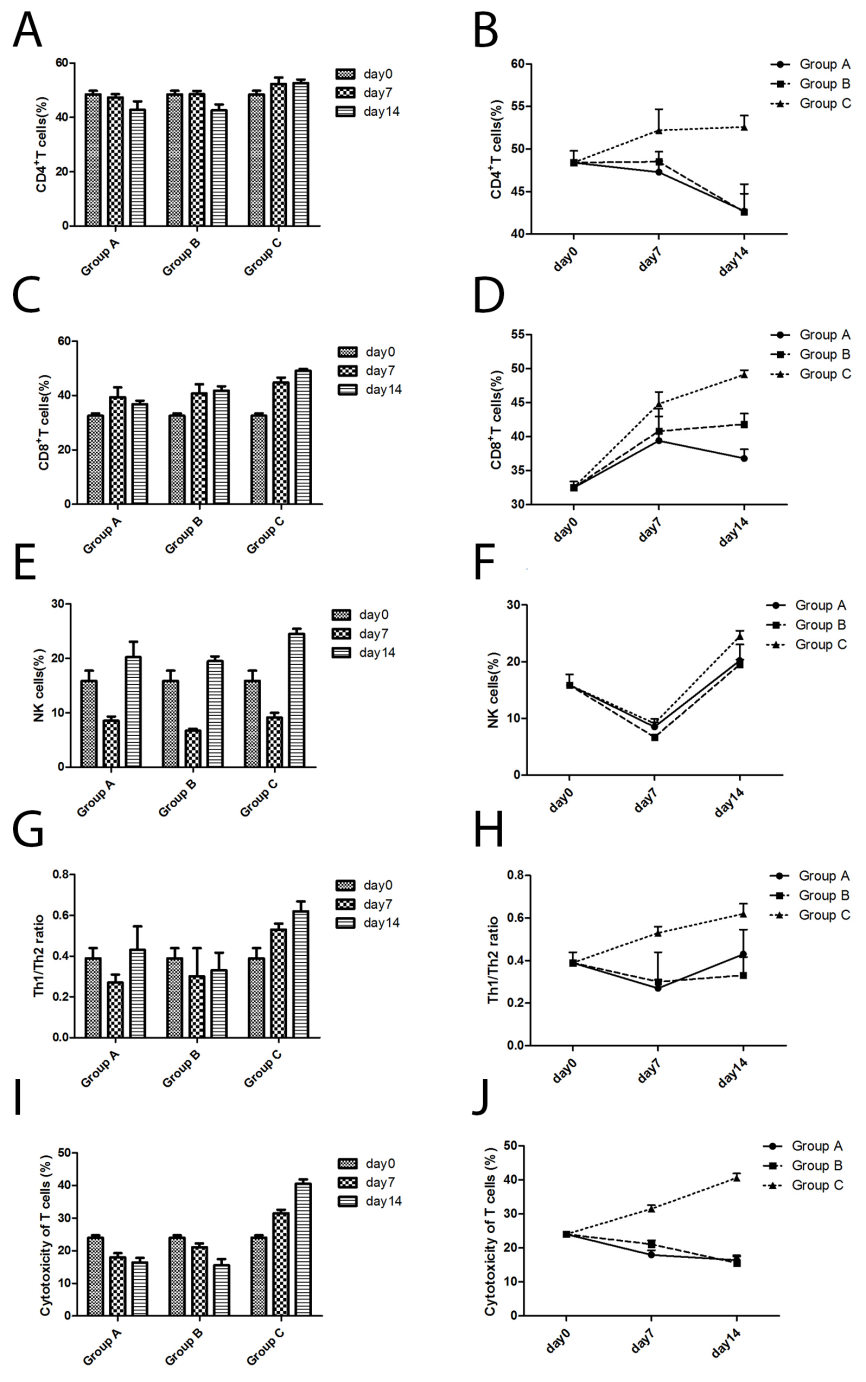
The changes in immune cells according to flow cytometric analyses of cells from three groups. **(A)** The CD4^+^ T cell numbers changed with time. **(B)** The differences in CD4^+^ T cell numbers among the three groups. **(C)** The CD8^+^ T cell numbers changed with time. **(D)** The differences in CD8^+^ T cell numbers among the three groups. **(E)** The NK cell numbers changed with time. **(F)** The differences in NK cell numbers among the three groups. **(G)** The Th1/Th2 ratio changed with time. **(H)** The differences in Th1/Th2 ratios among the three groups. **(I)** The cytotoxicity of T cells changed with time. **(J)** The differences in cytotoxicity of T cells among the three groups.

As time went on, the percentage of CD4^+^ T cells in Group C (Cryoablation group) showed an increasing trend, while that in Group A (Control group) and Group B (Surgery group) showed a declining trend. Compared to Group A (Control group) and Group B (Surgery group), the percentage of CD4^+^ T cells in Group C (Cryoablation group) increased significantly (*P* < 0.05, *P* < 0.05, respectively) on day 7 and day 14 (Figure 4A, 4B).

With time, the percent of CD8^+^ T cells in Group C (Cryoablation group) and Group B (Surgery group) manifested an increasing trend, while in Group A (Control group) a declining trend. Compared to Group A (Control group) and Group B (Surgery group), the percentage of CD8^+^ T cells in Group C (Cryoablation group) significantly increased (*P* < 0.05, *P* < 0.05, respectively) on day 7 and day 14 (Figure 4C, 4D).

As a function of time, the percentage of NK cells in all the three groups had the same trend: a decline at first, then an increase. Compared to Group B (Surgery group), the percentage of NK cells in the Control group and Cryoablation group increased slightly (*P* = 0.002, *P* < 0.001, respectively) on day 7. On day 14, the percentage of NK cells in the Cryoablation group increased significantly as compared to the Control group and Surgery group (*P* = 0.003, *P* = 0.001, respectively; Figure 4E, 4F).

### The T helper 1 (Th1)/Th2 ratio in the spleen

As time went on, ratio Th1/Th2 in Group C (Cryoablation group) showed an increasing trend, and Group A (Control group) and Group B (Surgery group) revealed a declining trend. Compared to Group A (Control group) and Group B (Surgery group), the Th1/Th2 ratio in Group C (Cryoablation group) significantly increased (*P* < 0.05, *P* < 0.05, respectively) on day 7 and day 14 (Figure 4G, 4H).

### Cytotoxicity against RM-1 cells according to an LDH assay

With time, the killing activity of cytotoxic T lymphocytes (CTLs) in Group C (Cryoablation group) manifested an increasing trend, while Group A (Control group) and Group B (Surgery group) showed a declining trend. Compared to Group A (Control group) and Group B (Surgery group), the killing activity of CTLs in Group C (Cryoablation group) significantly increased (*P* < 0.001, *P* < 0.001, respectively) on day 7 and day 14 (Figure 4I, 4J).

### Ki67 activity of tumors in the untreated region

**Figure 5:**
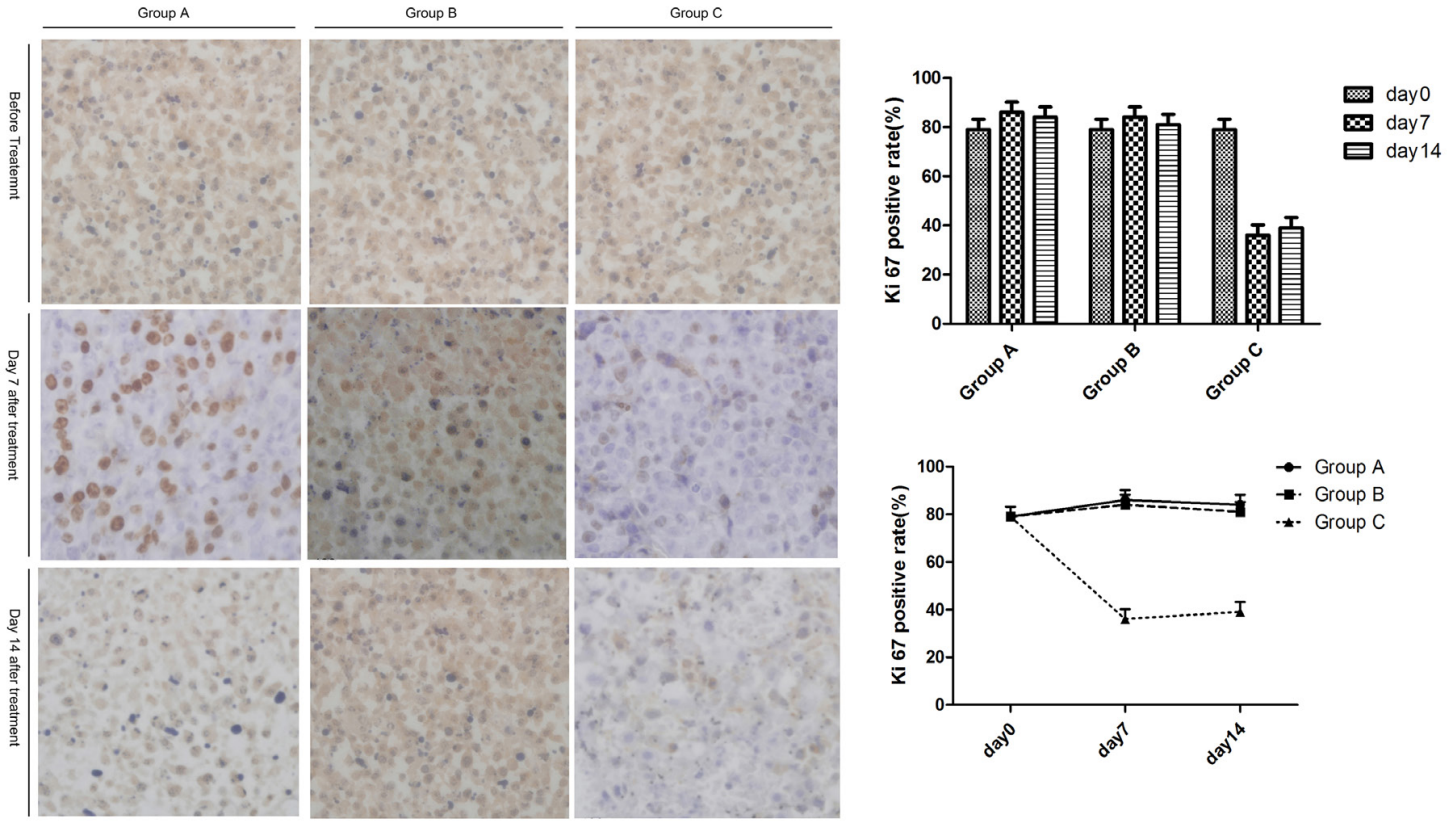
The expression of Ki67 in untreated tumors after treatment. The left panel shows the expression of Ki67 in untreated tumors on days 7 and 14. The right panel indicates the changes of Ki67 expression among the three groups with time.

As a function of time, the proliferation activity (Ki67) in untreated tumor tissue in Group C (Cryoablation group) showed a declining trend at first, then an increasing trend. Group A (Control group) and Group B (Surgery group) showed an increasing trend at first, then a declining trend. Compared to Group A (Control group) and Group B (Surgery group), the proliferation activity (Ki67) of the untreated tumor tissue in Group C (Cryoablation group) decreased significantly (*P* < 0.05, *P* < 0.05, respectively) on days 7 and 14. Compared to Group A (Control group) and Group B (Surgery group), there was no significant change in the other two groups (*P* > 0.05; Figure 5).

### Serum Hsp70 levels 

**Figure 6:**
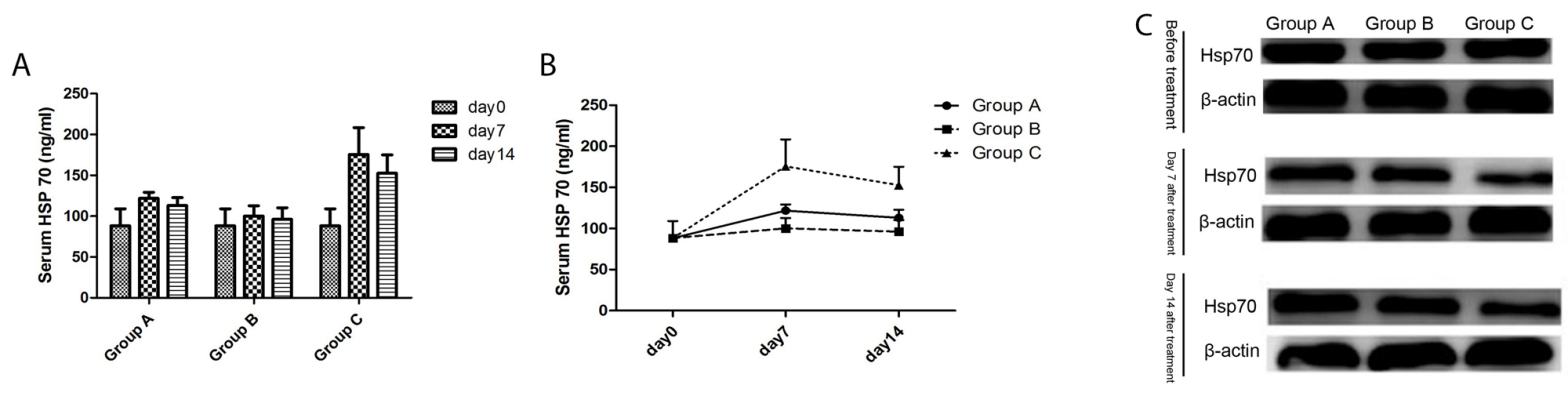
The Hsp70 levels of mice in three groups. **(A)** The serum Hsp70 level changed with time. **(B)** The differences in serum Hsp70 levels among the three groups. **(C)** The cytoplasmic Hsp70 level in untreated tumors on days 7 and 14 after treatment.

With time, the Hsp70 changes in the serum of all groups had the same trend: an increase at first, then a decline. Compared to Group A (Control group) and Group B (Surgery group), Hsp70 levels in the serum of group C (Cryoablation group) increased significantly (*P* < 0.01, *P* < 0.01, respectively) on days 7 and 14. There was no significant change in the other two groups (*P* > 0.05; Figure 6A, 6B).

### Hsp70 expression in the cytoplasm

As time went on, the expression of Hsp70 in Group C (Cryoablation Group) in the cytoplasm of tumor cells had a declining trend, while Group A (Control group) and Group B (Surgery group) manifested an upregulation trend. Compared to Group A (Control group) and Group B (Surgery group), the expression of Hsp70 in Group C (Cryoablation Group) in the cytoplasm of tumor cells significantly decreased on the 7th and 14th day (Figure 6C).

## DISCUSSION

As early as the 1970s [25], remission of metastases after cryosurgery in prostatic cancer (defined as an immunologic response) has been recognized. Bayjoo P and colleagues [26] have reported enhanced NK cytotoxicity following cryosurgery of an implanted liver tumor in rats. Hamad GG et al. [27] also demonstrated the transient immune response to cryosurgery in an experimental model. Cryoablated tumors in glioma mouse models induced an antitumor cellular immunological response, which increased the percentage of CD3^+^ T and CD4^+^ T cells in blood as was the case for NK cells [28]. During our clinical research, we also observed this phenomenon (Figure 1) induced by cryoablation of prostate cancer (defined as the abscopal effect).

Due to the complex environment in clinical settings, animal experiments have always been adopted for controlling the variables that affect the behavior of a biological system under study. Similarly, to detect the possible abscopal effects induced by cryoablation, we designed one bilateral subcutaneous xenograft tumor model, in which tumors in the right groin were designated as the primary endpoint and treated, while tumors in the left groin were regarded as the abscopal group and were left untreated. Hence, the dynamic growth of the untreated tumor in the left groin could strongly reflect the abscopal effect. Our experiments show that in the RM-1 prostate cancer model, local cryoablation treatment triggered systemic antitumor effects capable of controlling tumor growth at a remote site, consistent with the definition of the abscopal effect (Table 2). Cryoablation of a local site not only retarded the rate of untreated tumor growth, but also reduced the incidence of lung metastasis in mice and significantly prolonged the survival time of mice (Figure 2). It is likely that cryoablation for prostate cancer can induce the bystander effect. Based on evidence from previous studies [29, 30], cryoablation of RM-1 cells can elicit a protective immune response. Hence, we speculated that an immune response may contribute to the abscopal effect.

It is known that T cells and related cytokines play a major role in tumor immunity. Interferon γ (IFN-γ) is the primary cytokine that defines Th1 cells [31]: Th1 cells secrete IFN-γ, which in turn causes greater numbers of undifferentiated CD4^+^ cells (Th0) to differentiate into Th1 cells, representing a positive feedback loop while suppressing Th2 cell differentiation. Once antigen-specific immunity develops, CD8^+^ CTLs (effector T cells) release large amounts of IFN-γ, which promotes Th1 differentiation by upregulating the transcription factor T-bet, ultimately leading to cellular immunity. Otherwise, interleukin 4 (IL-4) is a cytokine that induces differentiation of naive helper T cells (Th0) into Th2 cells, which mediate immunosuppression [32]. In other words, Th1 cytokines appear to have a protective effect against tumor progression, whereas Th2 cytokines seem to favor tumor growth. The Th1/Th2 paradigm also provides the rationale for the development of tumors.

To evaluate the role of a cryoimmunological response after cryoablation, we compared the immune-cell numbers and cytokine levels of mice among three groups. Our study revealed the increasing trend of the spleen percentage of CD4^+^ T cells and CD8^+^ T cells, the Th1/Th2 ratio, the killing activity of CTLs after cryoablation on days 7 and 14 (*P* < 0.05), coupled with an increase of the spleen NK cell percentage in Group C (Cryoablation group), which suggested that cryoablation treatment can induce an antitumor immune response, which is mainly composed of CD8^+^ T cells, thereby inhibiting the growth of the untreated tumor, decreasing the rate of lung metastasis, and offering a survival benefit. In clinical settings, immune escape and persistently suppressed immune function contribute to the local recurrence or disease progression. Enhancing the immune function has certain clinical significance for improving the survival rate and quality of life. Our results proved that cryoablation for prostate cancer elicited the abscopal effect by triggering an immune response, which was similar to that in other local treatments [33]. Nevertheless, cryoablation alone was not sufficient for eliminating the abscopal tumor and inhibiting lung metastasis, suggesting that the degree of immunogenicity of a tumor may not be very strong. Other systemic treatment is needed to enhance the abscopal effect.

Additionally, multiple mechanisms may contribute to the abscopal effect. In our study, we tested whether the tumor proliferative activity could be employed to elicit an abscopal effect. Here, our data revealed a significant decline of proliferation activity (Ki67) of untreated tumor tissue in Group C. As a cellular marker for proliferation [34], the Ki-67 protein is strongly associated with cell proliferation, tumor invasion, metastasis, and survival [35, 36]. Higher expression of Ki-67 indicates a greater chance of proliferation, metastasis, and recurrence of cancer cells [37]. Clinical studies have shown that the protein expression of Ki-67 in lung cancer is related to the level of regulatory T (Treg) cells in peripheral blood. These data indicated that weak immune responses may facilitate the proliferation and metastasis of tumor cells. Similarly, an active immune response in our study was consistent with lower Ki-67 expression, indicating that the abscopal effect may be enhanced indirectly by the cell proliferation. Nevertheless, the underlying mechanism remains unclear.

Generally, the initiation, maintenance, and termination of an effective antitumor immune response requires a complex interplay between cellular (immune cells including effector and regulatory subsets) and humoral components (cytokines, chemokines, and antibodies)[21]. Various constitutive or inducible danger signals released by injured cells are known to play a determinant role in alerting the immune system about self-damage [21–23], e.g., high mobility box 1 protein (HMGB1) and heat shock protein 70 (Hsp70) may play a pivotal role in triggering antitumor immune responses [38, 39]. Our precious animal research experience [24] also revealed a rising HMGB1 level after cryoablation for prostate cancer, and the positive correlation between high HMGB1 levels and activated dendritic cells (DCs); this finding indicates that danger signals may be important for the cryoimmunological response. Apart from regulation of tumorigenesis [40], Hsp70 also regulates immune function, including antigen cross-presentation [41], dendritic cell maturation [42, 43], and NK cell [44] and myeloid derived suppressor cells (MDSC) activities [45]. In contrast to normal cells, tumor cells have higher basal Hsp70 and Hsp90 levels. Some stress factors, such as ionizing irradiation and thermal ablation always induce the synthesis, membrane transport, and release of Hsp70 or Hsp90[46]. Hence, their serum levels increased significantly after exposure to stressful conditions. Evidence indicatess that the function of Hsp70 varies according to the location: antiapoptotic effects and promotion of malignant transformation and distant metastasis in the cytoplasm [47, 48] but immune regulation in the membrane or extracellular site [49]. Our results showed a higher serum Hsp70 but lower cytoplasmic level after cryoablation, indicating that extracellular Hsp70 may also participate in immunoregulatory activities and in the abscopal effect. Similarly, other studies indicate that extracellular Hsp70, passively released from tumor cells or dying cells and actively trafficked via the endolysosomal pathway [50] also controls diverse immunoregulatory activities [51, 52]. Together with the above results, this evidence suggests that the inducible abscopal effect after cryoablation may be mediated by an immune response via the release of Hsp70. The possible mechanism is as follows: Hsp70-chaperoned peptides are recognized by CTLs after cross-presentation on major histocompatibility complex (MHC) class I molecules and thus generates a T-cell–mediated anticancer immune response.

## CONCLUSIONS

We found that the protective effect of cryoablation treatment against an abscopal tumor is mediated by an increase in the numbers of CD4^+^ T cells, CD8^+^ T cells, the Th1/Th2 ratio, and the killing activity of CTLs and NK cells. The underlying mechanism may contribute to the modulation of immune responses by promoting a release of Hsp70. The combination of weakened Ki67 activity and an activated immune response delayed spectator tumor growth, decreased the pulmonary metastasis rate, and prolonged animal survival, with an inducible abscopal effect. Nevertheless, our results confirmed a vital contribution of the immune response to the abscopal effect.

## MATERIALS AND METHODS

### Animals and experimental procedures

Six- to 8-week-old male BALB/c mice (live weight 18 to 25 g), free of mouse-specific pathogens, were obtained from the Institute of Laboratory Animal Science, Chinese Academy of Medical Sciences (Beijing, China). All the experimental procedures were approved by the ethics committee of Tiajin Medical University, Cancer Institute and Hospital. All surgical procedures were performed under anesthesia, and every effort was made to minimize the suffering.

The RM-1 prostate cancer cell line was purchased from Shanghai Institute of Life Sciences cell resource center, Chinese Academy of Medical Sciences (Beijing, China). RM-1 cells were cultured in the RPMI 1640 medium (SH30809.01B; HyClone) containing 10% of fetal bovine serum (SH30084.03; HyClone), 100 U/ml penicillin, 100 μg/ml streptomycin, and 50 μg/ml L-glutamine in a humidified incubator at 37°C with 5% CO_2_. RM-1 cells (5 × 10^6^) were intradermally injected into the right groin (targeted region) of each mouse. Three days letter, RM-1 cells (5 × 10^6^) were also intradermally injected into the left groin (untargeted region or abscopal region) of each mouse. When the diameter of the tumor in the right groin reached ~0.5 cm, mice (40 per group) were randomly assigned to receive a sham operation (Group A, Control group), surgery (Group B, Surgery group), or complete cryoablation (Group C, Cryoablation group). In total, ~60 mice were used in this study. All the mice were anesthetized by an intraperitoneal injection of a ketamine/xylazine mixture (23.75 mg/ml ketamine + 1.25 mg/ml xylazine; 100 μl per 25 g). The mice were prepped by shaving the targeted area and cleansing it with alternating 70% alcohol rinse and povidone iodine scrub before surgery. The mice of group A received a sham surgical operation. The skin above tumor was opened and then closed with a 4.0 surgical suture without resection. In group C, the skin above a tumor was opened via tumor resection, and then also closed with a 4.0 surgical suture. In group C, a 1.7-mm cryoablation probe (Endocare Per Cryo) was inserted into the tumor, and freezing was administered for 60–90 seconds under a working pressure of 17,225 kPa (2500 psi) and a temperature of -110°C to -125°C at the needle hub. Standard recovery procedures were implemented. All the above procedures in three groups were conducted only on tumors in the target region (right groin). The tumors in the untargeted region (left groin) received no treatment, only measurements and recording of the diameter of tumors with a vernier caliper. Survival rates were determined and recorded in 10 mice of each group. The remaining mice (10 per time point) received euthanasia before the procedures, 1 week, and 2 weeks later.

### Sample collection

Peripheral blood was collected by removing an eyeball before euthanasia. These blood samples were centrifuged at 3000 rpm for 5 minutes, and then serum samples were collected and stored at -20°C. Spleens, tumors in the left groin (untargeted tumors), and lungs were also collected. Lymphocytes from spleens were separated with a mouse lymphocyte separation medium (DKW33-R0100). Half of the untargeted tumors and lungs were fixed in a 4% formaldehyde solution for immunohistochemical staining, and the remaining untargeted tumors were stored in sterile storage tubes and frozen rapidly in liquid nitrogen.

### Flow cytometry

Lymphocytes from spleens were resuspended in staining buffer (PBS containing 3% of fetal bovine serum) and stained for 30 minutes at 4°C with an APC/Cy7-conjugated anti-mouse CD3 antibody (Lot. 100330, BioLegend), PE/Cy7-conjugated anti-mouse NK-1.1 antibody (Lot. 108714, BioLegend), APC-conjugated anti-mouse CD4 antibody (Lot. 100412, BioLegend), PerCP-conjugated anti-mouse CD8a antibody (Lot. 100732, BioLegend) as well as their corresponding isotype control antibodies as a negative control. Cells were fixed and permeabilized using the fix/perm kit (Lot. 88-8823-88, eBioscience). Intracellular IFN-γ and IL-4 staining was performed on the cells stained with a PE-conjugated anti-IL-4 antibody (Lot. 5014104, BioLegend) and FITC-conjugated anti-IFN-γ antibody (Lot. 505806, BioLegend) and its isotype control antibodies as a negative control. Flow cytometry was performed on a BD Canto II flow cytometer (BD Biosciences).

### Immunohistochemical staining

Tissue sections were immunostained with an anti-Ki-67 antibody (clone MIB-1; 1:100; Dako, Denmark). Tissue sections were deparaffinized in xylene, rehydrated through serial dilutions of ethanol, and washed in phosphate-buffered saline (pH 7.2), and then microwaved at 500 W for 2 × 5 min in 10 mM citrate buffer (pH 6.0). After rinsing in Tris-buffered saline (pH 7.6), the sections were immersed in a solution of H_2_O_2_ (3%) to block endogenous peroxidase activity, followed by the addition of 5% fetal calf serum (1:20 dilution). The samples were then incubated with a Ki-67 primary antibody (12202,1:400, Cell Signaling Technology [CST]) overnight at 4°C. After a wash, the primary antibody was detected by means of an appropriate secondary antibody (Universal PV9000 Kit; Zhongshan Biotechnology, Beijing, China), incubated for 30 min at 37°C, and then visualized with diaminobenzidine (DAB). Counterstaining was carried out with hematoxylin.

Briefly, the nuclear staining intensity was assessed in 1000 cells randomly selected from 5 areas (2 distinct lesions were selected in each area) by 2 of the authors and a pathologist, under a microscope at ×400 magnification, and were graded as follows: 0, negative <10%; 1+, weakly positive 10–33%; 2+, moderately positive 34–66%; and 3+, strongly positive >66%.

### Hematoxylin and eosin (HE) staining

Briefly, lung tissues from all the groups were sliced at 4 μm thickness in routinely processed paraffin blocks. These slices were deparaffinized, rehydrated, and rinsed in distilled water. Then, we added with hematoxylin with incubation for 5 min, and then performed color separation with alcohol containing 0.5% hydrochloric acid. Next, the slides were dyed with eosin, dehydrated with a gradient of ethanol, soaked in xylene, and mounted with neutral balsam.

### Heat shock protein 70 (Hsp70) detection

Total Hsp70 levels in serum samples of mice were measured using the Mouse Hsp70 ELISA Kit (Lot CSB-E08311m, Cusabio. China). Total protein was extracted from fresh tumor tissue according to protocols [53]. The protein concentration was determined based on a BCA standard curve (23225, Thermo Fisher). Western blotting was conducted for Hsp70 protein analysis, where the anti-Hsp70 antibody (4873, 1:1000, CST) was diluted 1:1000 and incubated overnight at 4°C in a sealed plastic box on a shaking device at low speed. The next day, the membrane was rinsed once in 1× Tris-buffered saline with Tween 20 (TBST) buffer for 15 min and twice for 5 min in fresh 1× TBST buffer to remove the unbound primary antibodies. After that, the membrane was incubated with a horseradish peroxidase–conjugated goat anti-rabbit antibody (#7077, CST) in a dilution of 1:2000 for 1 hour at room temperature. Next, the blot was washed three times for 5 min each with TBST. We removed the blot from the working solution and place it in a plastic sheet protector or clear plastic wrap. Pierce ECL Western Blotting Substrate (32106, Pierce) was used for protein detection.

### A cytotoxicity assay *in vitro*

Lymphocytes from spleens (effector cells) were incubated with RM-1 prostate cancer cells (target cells). Cytotoxicity was tested with a standard 4-hour CytoTox 96 non-radioactive cytotoxicity assay (G1780; Promega, USA) with a 40:1 effector cell-to-target cell ratio according to the manufacturer’s instructions.

### Statistical analysis

Statistical analyses were performed using the Statistical Package for Social Sciences (SPSS 13.0 for Windows; SPSS Inc., Chicago, IL). GraphPad Prism 5 software was used for graphics. Differences at each time point for each treatment group were evaluated via pairwise comparisons according to the mixed-effects analysis of variance (ANOVA) model. Data with *P* < 0.05 were considered statistically significant.
